# Differential Roles of Iron Storage Proteins in Maintaining the Iron Homeostasis in *Mycobacterium tuberculosis*

**DOI:** 10.1371/journal.pone.0169545

**Published:** 2017-01-06

**Authors:** Garima Khare, Prachi Nangpal, Anil K. Tyagi

**Affiliations:** 1 Department of Biochemistry, University of Delhi South Campus, Benito Juarez Road, New Delhi, India; 2 Vice Chancellor, Guru Gobind Singh Indraprastha University, Sector 16-C, Dwarka New Delhi, India; International Centre for Diarrhoeal Disease Research Bangladesh (icddr,b), BANGLADESH

## Abstract

Ferritins and bacterioferritins are iron storage proteins that represent key players in iron homeostasis. Several organisms possess both forms of ferritins, however, their relative physiological roles are less understood. *Mycobacterium tuberculosis* possesses both ferritin (BfrB) and bacterioferritin (BfrA), playing an essential role in its pathogenesis as reported by us earlier. This study provides insights into the role of these two proteins in iron homeostasis by employing *M*. *tuberculosis bfr* mutants. Our data suggests that BfrA is required for efficient utilization of stored iron under low iron conditions while BfrB plays a crucial role as the major defense protein under excessive iron conditions. We show that these two proteins provide protection against oxidative stress and hypoxia. Iron incorporation study showed that BfrB has higher capacity for storing iron than BfrA, which augurs well for efficient iron quenching under iron excess conditions. Moreover, iron release assay demonstrated that BfrA has 3 times superior ability to release stored iron emphasizing its requirement for efficient iron release under low iron conditions, facilitated by the presence of heme. Thus, for the first time, our observations suggest that the importance of BfrA or BfrB separately might vary depending upon the iron situation faced by the cell.

## Introduction

Iron is a vital element for the growth of animals, plants as well as microorganisms. It plays an important role in several biological processes and is essential for the activity of many enzymes involved in vital cellular functions ranging from respiration to DNA replication [1]. While the deficiency of iron is detrimental to cells, its excess is also potentially toxic as it promotes Fenton reaction resulting in the generation of highly toxic hydroxyl radicals [1]. Hence, the appropriate level of iron in the cell is maintained by its homeostasis based on the iron storage as well as acquisition pathways during its excessive availability and release for the cellular functions during its shortage. Most of the aerobic organisms possess ubiquitous iron storage proteins called ferritins which are highly symmetrical molecules comprising 24 subunits arranged in an octahedral pattern giving rise to an internal cavity capable of accommodating many thousands of iron atoms in a soluble non-toxic form which can be utilized by the cell during iron deficiency [2,3]. Ferritins have been further divided into three categories: (i) ferritins (Ftn) which contain a highly conserved ferroxidase centre and are best represented by eukaryotic H-chain ferritins and other similar proteins (ii) bacterioferritins (Bfr) which contain heme and are exclusively present in bacteria and (iii) Dps which are DNA binding proteins present in bacteria and archaea.

It has been reported in a number of organisms such as *Francisella tularensis*, *Histoplasma capsulatum* and *Salmonella typhimurium* etc. that the disruption of iron acquisition and storage genes renders the organism attenuated for its growth and virulence in the host [4, 5, 6].

Ferritins and bacterioferritins are known to be primarily associated with the maintenance of iron homeostasis, however, studies have also shown them to be involved in providing protection to cells against oxidative stress [7–12]. Although, a number of organisms are known to have both ferritins as well as bacterioferritins, their relative roles in maintaining iron homeostasis or in oxidative stress is variable. For instance, studies with a ferritin-deficient mutant of *Porphyromonas gingivalis* showed the importance of ferritin particularly for the survival of the bacterium under iron depleted conditions indicating that intracellular iron was stored in ferritin and was utilized under iron-restricted conditions [7]. However, the ferritin appeared not to contribute towards the protection of the organism against oxidative stress [7]. Moreover, *Escherichia coli* and *Campylobacter jejuni* are known to possess both ferritin and bacterioferritin wherein only ferritin has been reported to be important for their growth under low iron conditions while both ferritin and bacterioferritin mutations in these organisms produced an enhanced sensitivity to hydroperoxides [8,9]. Gonococcal Bfr, proposed to be composed of two different subunits BfrA and BfrB, serves as a source of iron during iron deprivation as well as provides protection to *Neisserria gonorrhoeae* against oxidative stress [10]. In the case of *Erwinia chrysanthemi* also, which has both the iron storage proteins, ferritin was found to be the main iron storage protein and exhibited an increased sensitivity to iron deficiency and to redox stress conditions [11]. Non heme-iron ferritin of *Helicobacter pylori* is involved in the formation of iron-containing subcellular structures which help in providing protection against iron overload [12].

*M*. *tuberculosis* encodes two ferritins–BfrA (Rv1876) and BfrB (Rv3841). However, *M*. *tuberculosis* does not possess the third form of ferritin i.e. Dps protein. While BfrA is a heme binding bacterioferritin, BfrB is a ferritin that does not bind heme. However, in view of the nomenclature assigned to them during the annotation of *M*. *tuberculosis* genome, both these proteins have continued to be addressed as Bfrs. By employing *M*. *tuberculosis* double mutant which was unable to synthesize BfrA and BfrB, we have earlier reported that these iron storing proteins are essential for the growth as well as pathogenesis of *M*. *tuberculosis* in guinea pigs [13]. Reddy *et al*. also showed that the double mutant lacking both BfrA and BfrB was growth defective under low iron conditions indicating the role of these proteins in iron homeostasis [13]. In *M*. *tuberculosis*, the synthesis of these proteins is regulated by IdeR (Iron dependent regulator) in response to varying levels of iron [14]. Recently, Pandey and Rodriguez made an attempt to determine the contribution of BfrA and BfrB in the maintenance of iron homeostasis of *M*. *tuberculosis* by employing single gene knockouts of *bfrA* and *bfrB* (15). It was reported that BfrB is required to (i) overcome iron limitation, (ii) provide tolerance to high iron and (iii) provide protection against oxidative stress to *M*. *tuberculosis* when cultured in various stressful conditions, whereas, BfrA was suggested to be dispensable for the iron homeostasis of *M*. *tuberculosis* [15]. However, Gold *et al*. earlier reported that *bfrA* gene has two promoters which are separately used to assure the transcription of this gene under low as well as high iron conditions suggesting its importance in the iron homeostasis of the cell [16]. Thus, in this study, we have employed Δ*bfrA* and Δ*bfrB* mutants of *M*. *tuberculosis* namely M.tbH37RvΔ*bfrA*, M.tbH37RvΔ*bfrB* and double knockout M.tbH37RvΔ*bfrA*Δ*bfrB* to analyze the role of these proteins in the physiology of *M*. *tuberculosis*. By studying the differential production of BfrA and BfrB under iron deficient and iron sufficient conditions, we have attempted to delineate the role of these proteins in iron homeostasis. We also demonstrate the importance of these proteins in conferring protection to the pathogen from iron-mediated oxidative stress. Moreover, for the first time, we establish the role of BfrA and BfrB in protecting *M*. *tuberculosis* under hypoxic conditions. We finally demonstrate that there are differences in the inherent iron storage as well as iron release capabilities of these two proteins.

## Materials and Methods

### Bacterial strains and media

Various *M*. *tuberculosis* H37Rv (M.tbH37Rv) strains: M.tb H37RvΔ*bfrA*, M.tbH37RvΔ*bfrB* and M.tbH37RvΔ*bfrA*Δ*bfrB* were maintained in Middlebrook 7H9 broth or 7H11 agar (Difco) supplemented with glycerol (0.5%), tween 80 (0.2%) and albumin-dextrose-catalase complex (ADC, 10%). Construction of various *M*. *tb* mutants have been previously reported [13]. Minimal media (MM) was employed to grow the cultures under defined iron conditions for the determination of growth curves and expression analysis. MM was prepared by adding asparagine (5 gm/litre), KH_2_PO_4_ (5 gm/litre), glycerol (2%), ZnCl_2_ (0.5 mg/liter), MnSO_4_ (0.1 mg/liter) and MgSO_4_ (40 mg/liter) in double distilled water (DDW). It was supplemented with tween 80 (0.2%) and albumin-dextrose (AD, 10%). DDW used for the preparation of media was treated with Chelex-100 resin to deplete it from iron. MM prepared by the above mentioned protocol is considered as low iron media containing <1μM iron [16].

### Growth conditions

All the experiments were performed by using frozen stocks of M.tbH37Rv, M.tbH37RvΔ*bfrA*, M.tbH37RvΔ*bfrB* and M.tbH37RvΔ*bfrA*Δ*bfrB* that were subcultured twice in MM containing Tween80 (0.2%) and AD (10%) to create iron deplete condition. For subculturing, various *M*. *tuberculosis* strains were grown in MB7H9 and were passaged twice in MM before inoculating for the growth curves (one passage comprised of the growth of the bacterium from the time of its inoculation at OD_600nm_ ~0.02 to its growth to reach stationary phase at OD_600nm_ ~3.0).

### Growth kinetics under various stress conditions

Growth under various stress conditions was monitored by inoculating the fresh medium (MM) to an OD_600nm_ of 0.02 (the inoculum was subcultured twice in MM to deplete iron). For carrying out the growth curves of M.tbH37Rv and the mutant strains under low and high iron conditions, FeCl_3_ was added to MM to reach a defined concentration and OD_600nm_ was monitored daily. For oxidative stress conditions, growth kinetics of M.tbH37Rv and the mutant strains was monitored in the presence of various concentrations of H_2_O_2_. These experiments were performed thrice and the mean values ± standard errors of the means are plotted.

### Growth kinetics under hypoxic conditions

Growth of M.tbH37Rv and the mutant strains was monitored under low oxygen tension. Briefly, the frozen stocks of *M*. *tb* strains were subcultured once in MB7H9 and the culture was then diluted to an OD_600nm_ of 0.02 in MB7H9 medium containing tween80 (0.2%) and AD (10%). 10 ml aliquot of this culture was dispensed in 15 ml screw capped tubes and left standing at 37°C incubator. At various time points, an aliquot of each strain was removed and the OD_600nm_ was measured to evaluate the growth of individual strains under low oxygen tension. Few 10 ml aliquots were also kept for shaking at 200 rpm from which an aliquot of each strain was removed and plated on MB7H11 agar to visualize any change in the growth of the strains. As a control, methylene blue was added to the cultures and disappearance of the blue colour marked the generation of hypoxic condition. These experiments were performed twice and the mean values ± standard errors of the means are plotted.

### Immunoblotting

Cellular levels of BfrA and BfrB in *M*.*tb* strains was analyzed under various stress conditions. Polyclonal antibodies against BfrA and BfrB of *M*. *tuberculosis* were prepared by immunizing rabbit with recombinant purified BfrA and BfrB. During the late log phase of the growth curve (~day 10), 20 ml culture was harvested. Lysates were prepared from the harvested cultures. Briefly, the pellet was resuspended in lysis buffer (20 mM Tris-HCl pH 8.0, 2 mM β-mercaptoethanol, 1 mM polymethylsulfonyl fluoride (PMSF)) and subjected to bead beating by using 0.1 mm zirconia beads. After 10 rounds (each round—5000 rpm, 3 minutes) of bead beating, samples were centrifuged at 13,000 rpm for 40 minutes. The supernatant was filtered through 0.2 μm filter and protein concentration was determined by using Bradford’s reagent [17]. 50 μg of lysate protein was loaded on SDS-polyacrylamide gel and subjected to immunoblotting by using anti-BfrA, and anti-BfrB rabbit polyclonal antisera. The lysates were also immunoblotted by using antibodies against SigA, which is one of the house-keeping genes of *M*. *tuberculosis*. Anti-SigA rabbit polyclonal antiserum was obtained from Dr. Jaya S. Tyagi laboratory (All India Institute of Medical Sciences, New Delhi, India). The intensity of the bands was quantified by using the Multi Gauge software (FUJIFILM Corporateion, Minato-ku, Tokyo, Japan).

### Site-directed mutagenesis

A site-directed mutant of BfrA was generated wherein methionine at position 52 was replaced with leucine (BfrAM52L). Mutagenesis was carried out by using XL-GOLD Quick Change kit (Stratagene, La Jolla, CA, USA) by employing appropriate primers (Forward primer: 5’-GGAGTCGTTCGACGAACTGCGGCACGCCGAGG-3’ and Reverse primer: 5’-CCTCGGCGTGCCGCAGTTCGTCGAACGACTCC-3’). The plasmid pASKiba43+*bfrA* (3.9 kb) containing the gene encoding BfrA was used as the template for mutagenesis. Mutagenesis was carried out as per the manufacturer’s recommendations and the resulting mutations were confirmed by DNA sequencing. *E*. *coli* BL21 (λDE3) cells were transformed with the recombinant plasmid. Transformed cells were grown in Luria Bertani medium containing ampicillin (50 μg/ml) at 37°C till an OD_600nm_ of 0.8–1.0 followed by induction with anhydrotetracycline (200 ng/ml) for 3 hours at 37°C.

### Purification of BfrA and BfrB

Transformed *E*. *coli* BL21 (λDE3) cells were induced as described above and the cells were harvested by centrifugation and resuspended in Tris.HCl pH 8.0 (20 mM) containing NaCl (50 mM), β-mercaptoethanol (2 mM) and PMSF (1 mM). Cells were disrupted by French Press followed by centrifugation at 16,000 rpm for 45 minutes at 4°C. The cell lysate was subjected to ammonium sulphate precipitation at 4°C followed by centrifugation at 13,000 rpm for 1 hour at 4°C. In the case of BfrB, purification was carried out as described earlier [3]. For BfrA and BfrAM52L, 25–40% ammonium sulphate precipitate suspended in 5 ml of 20 mM Tris HCl pH 8.0 containing 100 mM NaCl (buffer A) was loaded onto a Sephacryl S-300 column (2.5 cm× 92 cm) pre-equilibrated with buffer A and developed at a flow rate of 0.5 ml/min. 5 ml fractions were collected and analyzed by electrophoresis by using a 12.5% SDS-polyacrylamide gel for purity. Fractions containing BfrA protein were further purified by using a Resource Q (6 ml) column on AKTA purifier. Fractions were analyzed by electrophoresis on a 12.5% SDS-polyacrylamide gel for purity and the fractions containing BfrA were pooled and dialyzed against 20 mM Tris HCl, 100 mM NaCl pH 8.0. In addition, both the purified proteins (BfrA and BfrB) were analysed by FPLC by using 3 ml Superdex G-200 (5 mm x 150 mm) analytical column to verify the purity of the proteins. The FPLC profile showed predominantly a single peak for both the proteins, further confirming the purity of both these purified proteins (data not shown). Protein concentration was determined by using Bradford’s reagent with bovine serum albumin as the standard [17]. Heme contents were determined by measuring the intensity of heme (Soret peak) at 418 nm.

### In vitro iron incorporation assay

0.25 μM of BfrA or BfrB was incubated with varying concentrations of ammonium ferrous sulphate (freshly prepared in 0.015 N HCl) in HEPES (0.1 M), pH 6.5 in a reaction volume of 1 ml for 2 hours at RT. The reaction mix was incubated for 2 hours at RT allowing Fe(II) ions to get oxidized and subsequently get incorporated as Fe(III) ions in the protein shell. The free Fe(II) in the solution was determined by the addition of ferrozine reagent (1 mM) to the supernatant obtained after centrifugation of the samples and absorbance of the ferrozine-Fe(II) complex was measured at 570 nm. The absorbance for each sample was normalized with the negative control (buffer plus AFS) values to take into account the auto-oxidation of ferrous ions under these reaction conditions. The binding capacity is represented as the percentage of iron bound to the protein at various iron:protein ratios and is defined as the maximum iron:protein ratio at which 100% iron is bound by the protein.

### Kinetic measurements of iron release

0.25 μM protein was mineralized with ammonium ferrous sulphate (125 μM) in 0.1 M HEPES, pH 6.5, followed by incubation at room temperature for 2 hours. The incorporation of iron in each protein was monitored by measuring the enhancement in the absorbance at 310 nm. Iron release was initiated by the addition of sodium ascorbate (250 mM) in the presence of ferrozine reagent (1 mM) prepared in distilled water. The quantity of released iron was measured kinetically by monitoring the absorbance of the Fe(II)-Ferrozine complex at 570 nm.

## Results

### Differences in BfrA and BfrB levels during the growth of *M*. *tuberculosis* under various iron concentrations

**Fig 1:**
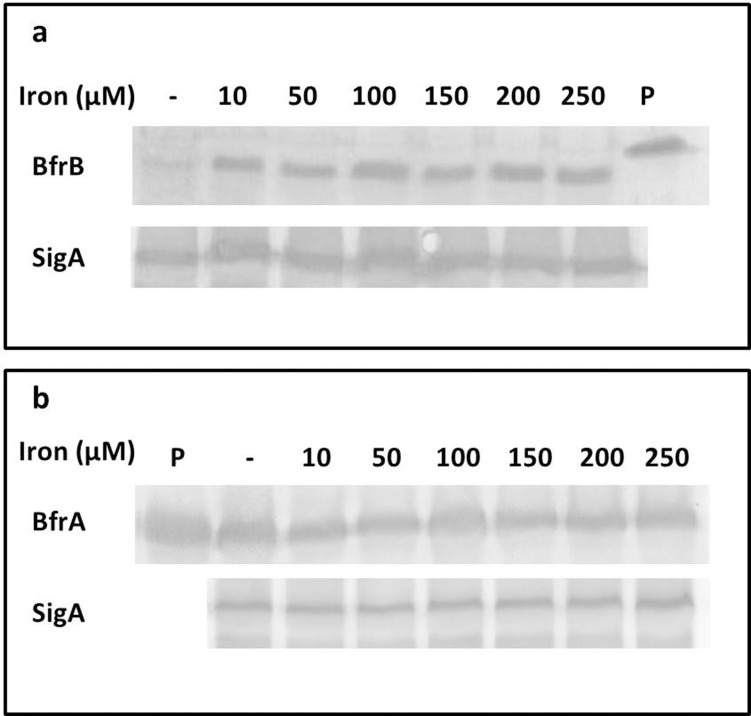
Iron mediated alterations in the levels of *M*. *tuberculosis* ferritins. *M*. *tuberculosis* H37Rv strain was grown in the minimal media either without iron (-) or with increasing iron concentrations (ranging from 10 μM to 250 μM). 50 μg of the lysate protein was subjected to 15% SDS-PAGE followed by immunoblotting by using antibodies against (a) BfrB and (b) BfrA of *M*. *tuberculosis*. For loading control, the same lysates were also immunoblotted by using antibodies against SigA. The immunoblot shown is representative of one experiment, however, the same pattern was observed across three experiments. P: Purified protein (positive control).

We studied the cellular levels of *M*. *tuberculosis* BfrA and BfrB during the growth of the pathogen under various iron concentrations ranging from iron starvation to iron rich conditions (≥ 10 μM) to find out if these proteins are produced differentially under varying iron concentrations by measuring the relative distribution of BfrA and BfrB under these conditions [18, 19, 20]. It was observed that under iron starved conditions, there was very little production of BfrB, however, the addition of 10 μM iron to the growth medium quickly induced its synthesis by 1.5 times (Fig 1A). Additional increase in the amount of iron in the medium did not further induce the synthesis of BfrB in the cell. In contrast, when the levels of BfrA protein were evaluated under different concentrations of iron in the growth medium, it was observed that the synthesis of BfrA protein did not seem to respond to the concentration of iron in the medium as addition of iron to the medium at different concentrations did not influence the level of BfrA in the cell (Fig 1B). These observations suggested the possible role of BfrA, the bacterioferritin homologue, in maintaining the basal iron homeostasis of the cells, whereas the ferritin analogue BfrB is possibly required for the quenching of excess iron to protect the cell from the iron-mediated toxicity.

Further, to check the specificities of the anti-BfrA and anti-BfrB polyclonal antisera, we carried out immunoblot analysis of the cell lysates derived from Δ*bfrA* mutant and Δ*bfrB* mutant. Our results demonstrated that the anti-BfrA antiserum did not cross react with BfrB since no band was detected at 20 kDa in the Δ*bfrA* mutant derived cell lysate. However, BfrA was recognized in cell lysates obtained from *M*. *tuberculosis* H37Rv as well as the Δ*bfrB* mutant. Similarly, anti-BfrB antiserum did not cross react with BfrA in the cell lysate derived from Δ*bfrB* mutant (S1 Fig).

### Role of BfrA and BfrB in providing protection to *M*. *tuberculosis* under stressful conditions

**Fig 2:**
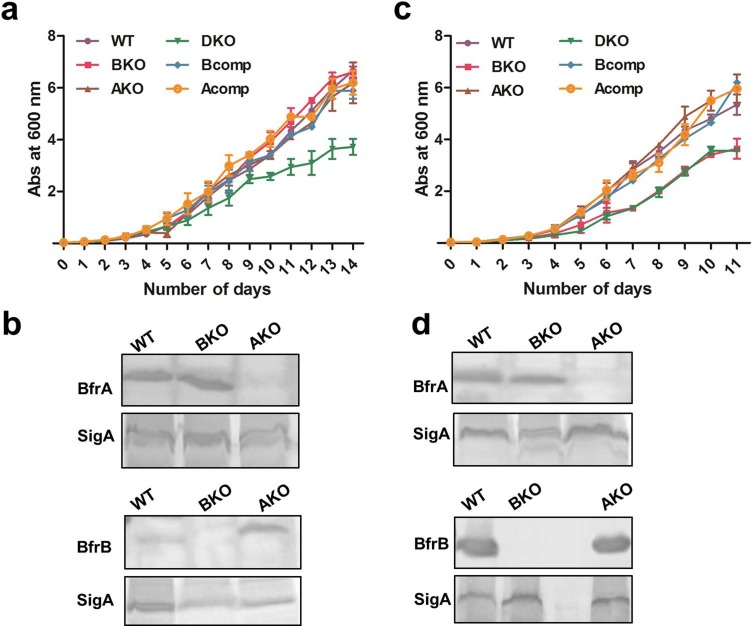
Effect of Bfr deletion on the growth of *M*. *tuberculosis* under iron starved and iron rich conditions. Various *M*. *tuberculosis* strains were grown in MB7H9 and were passaged twice in MM before inoculating for the growth curves (one passage comprised of the growth of the bacterium from the time of its inoculation at OD_600nm_ ~0.02 to its growth to reach stationary phase at OD_600nm_ ~3.0). Subsequently, growth of various *M*. *tuberculosis* strains was monitored at indicated time points under various conditions (a) iron starvation and (c) iron excess (250 μM FeCl_3_). These experiments were performed thrice and the mean values ± standard errors of the means are plotted. Immunoblotting was performed by using antibodies against BfrA (upper panel) and BfrB (lower panel) in the lysates of *M*. *tuberculosis* cells grown under (b) iron starvation and (d) iron excess (250 μM FeCl_3_). For internal control, the same lysates were immunoblotted by using antibodies against SigA. The immunoblot shown is representative of one experiment, however, the same pattern was observed across three experiments. WT: M.tbH37Rv, BKO: Δ*bfrB* mutant, AKO: Δ *bfrA* mutant, DKO: Δ*bfrA* and Δ*bfrB* double mutant, Bcomp: Δ*bfrB* mutant complemented with *bfrB* gene and Acomp: Δ*bfrA* mutant complemented with *bfrA* gene.

The ferritins or bacterioferritins store excess iron to prevent cells from iron toxicity and release the stored iron during low iron supply. The influence of the disruption of these genes on the viability of the cell was studied under variable iron conditions by employing the mutant strains. It was observed that when the cells were subjected to low iron minimal medium, the absence of either BfrA or BfrB separately did not influence the growth of the pathogen, while the double knockout (lacking both BfrA and BfrB) exhibited a growth defect as compared to the single mutants (Fig 2A). These results are at variance in comparison to the observations reported by Pandey and Rodriguez, wherein they showed that Δ*bfrB* mutant was found to be growth defective under low iron conditions [15]. Our observations suggested that both BfrA and BfrB provide the necessary iron to the cell in iron deprived conditions and the iron stored in either of the ferritins (BfrA or BfrB) can be utilized by the cell for normal growth during iron starvation. To understand the role of these proteins in iron homeostasis, immunoblot analysis was carried out by employing the cell lysates from Δ*bfrA* and Δ*bfrB* mutants separately (Fig 2B). It was observed that the BfrB levels were elevated by 1.5 times in Δ*bfrA* mutant, suggesting its compensatory role in providing iron to the cell under iron limiting conditions (Fig 2B). However, there was no change in the cellular levels of BfrA in the Δ*bfrB* mutant as observed by the immunoblot anaylsis (Fig 2B) indicating that existing BfrA levels in this case were sufficient to provide necessary iron suggesting a possible role for BfrA in maintaining iron homeostasis under low iron conditions.

As iron storage proteins are known to protect cells from iron-mediated toxicity, we also measured the growth of the parental and mutant strains in iron excess conditions. It was observed that Δ*bfrB* mutant and the double mutant were severely compromised in their ability to survive under high iron conditions (250 μM FeCl_3_), however, Δ*bfrA* mutant was unaffected by the iron overload in comparison to the parental strain (Fig 2C). Complementation of M.tbH37RvΔ*bfrB* with a functional copy of *bfrB* gene reverted the growth defect observed in the Δ*bfrB* mutant. These results correlate with the earlier studies wherein the investigators had observed that Δ*bfrB* single mutant was compromised in its growth in high iron conditions in comparison to the parental strain [15]. The growth defect observed in case of the Δ*bfrB* mutant and the double mutant lacking both these proteins was similar when subjected to iron excess conditions, implying that BfrB acts as the major defense protein against iron toxicity while BfrA does not play an important role in quenching the excess iron. In addition, the levels of BfrB remain unaltered in the Δ*bfrA* mutant indicating that the existing BfrB level is sufficient to prevent the cells from iron-mediated toxicity (Fig 2D). Thus, our observations suggest that the importance of BfrA or BfrB separately might vary depending upon the iron situation faced by the cell.

### Role of BfrA and BfrB in protecting the cells from oxidative stress

**Fig 3:**
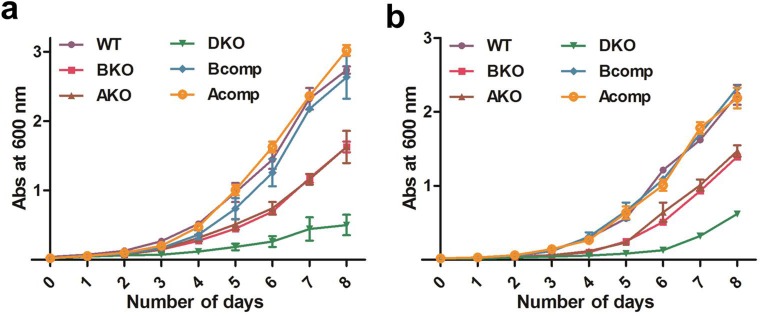
Role of ferritins in providing protection to *M*. *tuberculosis* against oxidative stress. Various *M*. *tuberculosis* strains were grown in the minimal media containing (a) 1 mM H_2_O_2_ and (b) 50 μM FeCl_3_ + 0.25 mM H_2_O_2_. These experiments were performed thrice and the mean values ± standard errors of the means are plotted. WT: M.tbH37Rv, BKO: Δ*bfrB* mutant, AKO: Δ *bfrA* mutant, DKO: Δ*bfrA* and Δ*bfrB* double mutant, Bcomp: Δ*bfrB* mutant complemented with *bfrB* gene and Acomp: Δ*bfrA* mutant complemented with *bfrA* gene.

It has been earlier reported in many organisms that iron storage proteins provide protection to the cells from oxidative stress, however, the role of ferritins and bacterioferritins in different organisms is at variance [7, 8, 10, 21]. Hence, the role of BfrA and BfrB was evaluated in providing protection to *M*. *tuberculosis* against exposure to hydrogen peroxide. It was seen that both the single mutants were more susceptible to the oxidative stress (1 mM H_2_O_2_) in comparison to the parental and complemented strains while the deletion of both the genes severely impaired the growth (Fig 3A). These observations suggested the role of both the iron storage proteins (BfrA and BfrB) in providing protection to the pathogen from oxidative stress. Effect of oxidative stress is known to get aggravated in the presence of free intracellular iron due to Fenton reaction which leads to the formation of hydroxyl radicals and insoluble ferric aggregates leading to increased cellular death [22]. It is expected that the presence of functional ferritins would help the cells survive against iron-mediated oxidative stress by depleting the free iron pool. Hence, we assessed the sensitivity of the mutant strains to hydrogen peroxide treatment in the presence of iron. Addition of 1 mM H_2_O_2_ inhibited growth of the parental cells in the presence of 50 μM FeCl_3_ suggesting the impact of iron-mediated toxicity on cells, hence, we employed a lower concentration of H_2_O_2_ (0.25 mM) which was tolerated by the parental strain (S2 Fig). The growth of the Δ*bfrA* and Δ*bfrB* double mutant was, however, severely compromised in comparison to the parental strain when the cells were treated with 0.25 mM H_2_O_2_ in the presence of 50 μM FeCl_3_ whereas the single mutants were also impaired though to a lesser extent (Fig 3B). It is to be noted that both the proteins contribute equally to this protection independently and the presence of iron causes much more pronounced effect on the susceptibility of the mutant strains to oxidative damage, emphasizing the role of both these proteins in providing protection against iron-mediated oxidative toxicity.

### Ferritins impart protection to *M*. *tuberculosis* under hypoxic conditions

**Fig 4:**
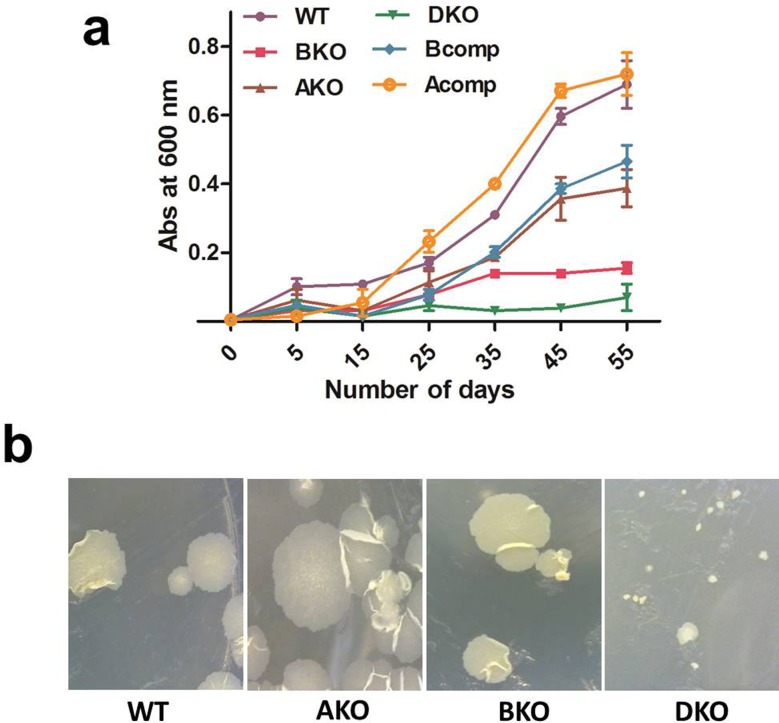
Deletion of ferritins results in diminished survival of *M*. *tuberculosis* under hypoxic conditions. *M*. *tuberculosis* strains were grown in MB7H9 media in screw capped tight 15 ml falcons as 10 ml aliquots. (a) The aliquots of each strain were kept at 37°C standing and the growth of various strains was monitored by measuring the absorbance at 600 nm at various time points. (b) Few aliquots of each strain were also kept at shaking at 200 rpm. An aliquot of each strain was removed and plated on MB7H11 agar to visualize any change in the growth of the strains. These experiments were performed twice and the mean values ± standard errors of the means are plotted. WT: M.tbH37Rv, BKO: Δ*bfrB* mutant, AKO: Δ *bfrA* mutant, DKO: Δ*bfrA* and Δ*bfrB* double mutant, Bcomp: Δ*bfrB* mutant complemented with *bfrB* gene and Acomp: Δ*bfrA* mutant complemented with *bfrA* gene.

While there are reports that *M*. *tuberculosis* BfrB is upregulated under hypoxic conditions [23, 24], its repercussions have not been dealt with in any study published so far. Hypoxia is a condition often faced by the pathogen during progression of the disease [25], thus its physiological relevance is obvious and for this reason we were keen to study what role, if any, BfrB plays when the pathogen is exposed to hypoxic conditions. We grew the pathogen under hypoxic conditions as described earlier [26] and studied the growth patterns of all three mutants of *M*. *tuberculosis* by comparing them with the parental strain. In order to relate our observations exclusively to hypoxic conditions, these studies were carried out in MB7H9 medium which provides sufficient iron for the growth of the mutants as well as the parental strain. It was observed that the Δ*bfrA* and Δ*bfrB* double mutant did not grow under hypoxic conditions, whereas the Δ*bfrB* mutant alone presented a strong growth defect (Fig 4A). The Δ*bfrA* mutant also displayed a growth defect though it was much less pronounced than in the case of the Δ*bfrB* mutant or the double mutant (Fig 4A). The defect seen in the case of both single knockout mutants was complemented by introducing *bfrA* and *bfrB* genes in their respective mutants although we observed partial complementation in the *bfrB* complemented strain (discussed later). Thus, our results suggest that these proteins play an important role in preventing the pathogen from the damage caused by hypoxic conditions. Further, when the cells grown under hypoxic conditions were analyzed for their growth on MB7H11 agar media, the colonies of the double mutant were rather small and grew very slowly in comparison to the parental strain, however, both the single mutants i.e. Δ*bfrA* mutant and Δ*bfrB* mutant showed normal colony size with the growth that was comparable to the parental strain (Fig 4B).

### Difference in the iron storage and release capacities of BfrA and BfrB

**Fig 5:**
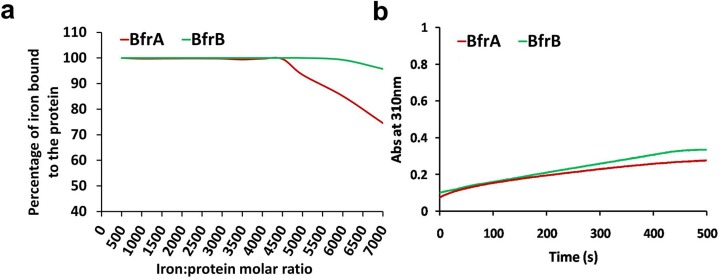
Iron storage capacities of BfrA and BfrB. Purified proteins were incubated separately with increasing amounts of iron for 2 hours at room temperature to facilitate the storage of iron as ferric mineral and determine the iron storing capacity of the protein. The residual unbound excess iron was detected by the addition of ferrozine reagent which complexes with free ferrous present in the solution forming a purple coloured product that is analyzed at 570 nm. (a) The figure depicts the percentage of iron incorporated by the protein at increasing iron:protein molar ratios. (b) Rate of iron oxidation was monitored by measuring the absorbance at 310 nm by incubating protein and iron in a ratio of 1:500. Rate was calculated as change in absorbance per unit time for both the proteins and was found to be similar. These experiments were performed twice, however, the figure shown is representative of one experiment.

To get more detailed information on the functions of BfrA and BfrB, we biochemically characterized these two proteins. When the storing capacity of these proteins was evaluated against increasing concentrations of iron (iron: protein molar ratio ranging from 500 to 7000), it was observed that BfrB has a higher capacity to store iron molecules than BfrA as can be seen in Fig 5A wherein BfrA was able to store upto 4500 molecules of iron/protein whereas BfrB could take upto 6000 iron molecules/protein. An alternative explanation to this observation could be provided by differential uptake efficiency of iron by these two proteins. Hence, we analyzed the rate of uptake of iron by BfrA and BfrB. However, our results clearly indicated that this differential storage of iron by these two proteins was not due to differential uptake rates as both the proteins took up iron at the same rate (Fig 5B). These observations justify cell’s preference to choose BfrB as preferred protein for iron storage under high iron conditions faced by the pathogen.

**Fig 6:**
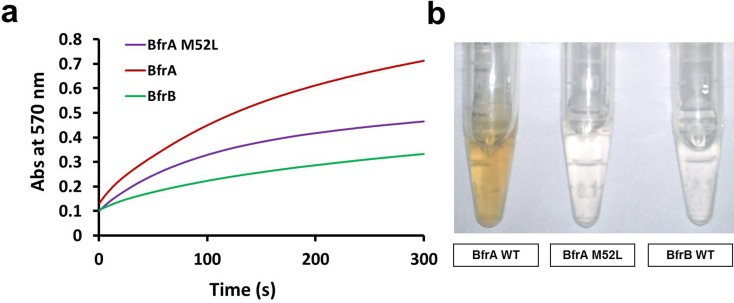
Iron release capacities of BfrA and BfrB. Purified proteins (0.1 μM) were incubated separately with 125 μM of iron for 2 hours at room temperature to facilitate the formation of holo-proteins. (a) Iron release was initiated by incubating the holo-proteins with sodium ascorbate and ferrozine and rate of iron release was monitored by measuring the absorbance of the Fe(II)-Ferrozine complex at 570 nm. (b) Figure shows the characteristic brown colour in BfrAWT(1mg/ml) due to the presence of heme whereas BfrAM52L (1 mg/ml) appears similar to BfrBWT (1 mg/ml) due to absence of heme. These experiments were performed twice, however, the figure shown is representative of one experiment.

From our results, it appeared that while BfrB can act as a preferred iron storing protein under high iron conditions due to its more efficient iron storing properties, BfrA could be preferred to mediate iron homeostasis under low iron conditions. This led us to think whether BfrA could be chosen for this purpose possibly due to its more efficient iron release properties as compared to BfrB. Thus, we examined the difference in the iron release properties of both these proteins. The purification of parental BfrA protein (BfrAWT) was carried out as described in the material and methods and the purity was analysed by SDS-PAGE (S3 Fig). Purification of parental BfrB protein (BfrBWT) was carried out as described previously [3]. It was observed that the iron release in the case of BfrA was 3 times faster in comparison to BfrB (Fig 6A), thus making it a protein of choice for iron release under low iron conditions. Besides, BfrA is a heme binding protein and heme is often reported to be associated with the iron release process from the bacterioferritins [27, 28]. Met52 is a conserved residue which is known to be involved in heme binding in bacterioferritins [27, 28, 29]. Hence, we also generated a site directed mutant of BfrA wherein Met at position 52 was replaced with leucine (BfrAM52L). The purification of BfrAM52L was carried out similarly as BfrAWT and the purity was analysed by SDS-PAGE (S3 Fig). The mutation at Met52 position resulted in a heme-free BfrA variant as visualized by the loss of its characteristic red colour (Fig 6B). This was validated by measuring the heme content which was found to be undetectable in BfrAM52L as compared to 6 heme molecules/protein in the parental molecule (Heme contents were determined by measuring the intensity of heme (Soret peak) at 418 nm with ε_418 nm_ = 107,000 M^−1^ cm^−1^) (data not shown). The removal of heme from BfrA resulted in more than 1.5 times reduction in its iron release capacity demonstrating thereby that heme could be playing an important role in the process (Fig 6A).

## Discussion

In a pathogen such as *M*. *tuberculosis*, iron homeostasis plays a vital role. Iron storage proteins such as ferritins and bacterioferritins play an important role in maintaining cellular iron homeostasis. Some organisms possess only one of the two iron storage proteins, while others are known to posses both bacterioferritins as well as ferritins. The major function of these proteins is to store excess iron in iron rich conditions and release it in the times of iron starvation. However, what is not clearly understood is the relative importance of these two classes of ferritins when present within the same organism. Has the cell assigned distinct task to these proteins to perform differential functions? How does the cell benefit from the presence of two iron storage proteins? *M*. *tuberculosis* also possesses two iron storing proteins, one of them being heme bound bacterioferritin (BfrA) and the other one being a non-heme binding ferritin (BfrB). In this study, we have ventured to identify the relative importance of these two proteins in the physiology of this important pathogen by employing *M*. *tuberculosis* mutants of these proteins.

Our studies on the production of these proteins under various concentrations of iron in the cell show that while the production of BfrA remains almost constant irrespective of iron levels, BfrB production is sensitive to iron levels the cell is exposed to, as BfrB production was induced as a response of the cell to the iron increment (Fig 1). It is worth mentioning that the production of BfrA in *M*. *tuberculosis* is regulated by two different promoters, which are sensitive towards iron levels in an opposite manner [16]. One of the promoters P_low_ responds to low levels of the iron while the other promoter i.e. P_high_ is switched on at high iron concentrations [16]. The differential production of these iron storing proteins in response to varying concentrations of iron suggests that these proteins could play different roles in the iron homeostasis exercised by the pathogen. The fact that the pathogen has evolved two different promoters for BfrA to maintain its level in the cell irrespective of iron concentrations suggest that BfrA might play a key role in the homeostasis of iron in the cell. In contrast, the increase in BfrB levels in response to high cellular iron points out its role in providing protection to the cell against iron-mediated oxidative damage by storing the excess iron.

Analysis of the growth of various mutants and their comparison with the wild type *M*. *tuberculosis* was carried out by cultivating the cells under varying iron conditions. Our results demonstrated that although simultaneous loss of both the proteins rendered the pathogen unable to grow under low iron conditions, lack of a single protein (BfrA or BfrB) did not affect growth of the pathogen which apparently suggested that the absence of any one of these proteins could be compensated by the presence of the other protein (Fig 2A). The immunoblot studies provided further insights into their role. Similar levels of BfrA protein in the Δ*bfrB* mutant as well as the parental strain suggested that BfrA at its normal level could suffice and compensate for the absence of BfrB and support normal growth of the mutant in the absence of BfrB. However, in case of the Δ*bfrA* mutant, the level of BfrB was induced 1.5 times in comparison to its level in the parental strain suggesting thereby that unlike BfrA, BfrB at its normal level was unable to support normal growth in the absence of BfrA and its level has to be induced to compensate for the loss of BfrA. This further corroborates that BfrA is an important protein for the growth of the pathogen under low iron conditions (Fig 2B), which may help the pathogen by quick release of the iron under low iron conditions. Its proposed role is further supported by the presence of heme in the protein, as heme is known to accelerate the release of iron by reducing the stored ferric molecules into ferrous counterparts, thus making them available for cellular functions [28]. In the case of *P*. *aeruginosa*, Bfd proteins which are known to be upregulated under low iron conditions, are reported to help heme mediated iron release [30, 31, 32]. *M*. *tuberculosis* also has *bfd* gene placed divergent to *bfrA* gene and its expression is regulated by IdeR [16]. Although, its upregulation under low iron conditions is reported [16], its participation in the release of iron under these conditions is still a matter of speculation.

*M*. *tuberculosis* has evolved several mechanisms to subvert the hostile environment that it faces with in the host including phagosomal arrest, prevention of acidification of the phagosomal compartment and protection against oxidative damage. In the phagosomes, it is exposed to myriad of host defense molecules like oxygen and nitrogen radicals (oxidative stress). Thus, we wanted to evaluate the role of BfrA and BfrB in providing protection against the oxidative stress in both low and high iron conditions by monitoring the growth profiles of the mutants and the parental strain in *in vitro* broth culture. It is known that superoxide radicals generated by various sources in the cell lead to reductive leaching of iron molecules from iron bound proteins having oxidatively labile iron [33]. This release of iron molecules in the cellular milieu can further undergo Fenton reaction in the presence of hydrogen peroxide leading to the formation of insoluble ferric ions and hydroxyl radicals and, hence, cause toxicity to the cell. To avoid this damage, the pathogen utilizes these iron storage proteins that help in quenching the released iron ions. Our growth kinetics data on various *M*. *tuberculosis* strains, also suggested this phenomenon as the double mutant displayed a highly compromised growth as compared to the parental strain whereas the single mutants also showed a marked defect in the growth when the cells were exposed to 1 mM H_2_O_2_. Additionally, oxidative stress is known to get aggravated under high iron condition [22]. As expected, it was seen that addition of iron to the medium containing H_2_O_2_ resulted in severely compromised growth of the double mutant as well as both the single mutants (Fig 3B). Thus, our data suggest a crucial role of these proteins in safe guarding the pathogen from the iron-mediated oxidative damage.

BfrB has been known to be upregulated when *M*. *tuberculosis* is subjected to hypoxia [23, 24], however, its implication in the survival of the pathogen, if any, has not been studied. Our studies on the growth of various *bfr* mutants and the parental strain under hypoxic conditions show that the double mutant lacking both the proteins did not survive under hypoxic stress and the Δ*bfrB* mutant was also highly compromised for growth in comparison to the parental strain. The Δ*bfrA* mutant also exhibited unfavorable growth influence when exposed to hypoxia but the influence on this mutant in comparison to the Δ*bfrB* mutant was much less pronounced (Fig 4A). The growth defect observed in the case of the Δ*bfrA* mutant was fully complemented on introduction of the *bfrA* gene, however, complementation of the Δ*bfrB* mutant with the *bfrB* gene showed partial complementation. This is often observed when a gene is complemented on a plasmid. The probable reason for this could vary. For example the expression of a gene in different contexts (in the chromosome versus in the plasmid) can be different. More importantly, in this particular case, it could be related to the extended duration of these growth curves (~60 days). The complemented strain was generated by electroporation of the mutant strain with the plasmid containing the wild type copy of the gene and chloramphenicol antibiotic resistance gene as the selection marker. However, all these growth kinetic experiments have been performed in the absence of any antibiotic selection pressure and thus it is possible that if the complemented strain is grown for a long period without antibiotic, the plasmid may get lost resulting in partial complementation. However, further investigations will be required to validate this phenomenon. It may be added that the complementation in case of the Δ*bfrA* mutant was complete possibly because the growth defect observed in the case of this mutant was not as much pronounced as it was for the Δ*bfrB* mutant. In addition, it is known that in case of oligodendrocytes and alveolar cells, ferritins are upregulated in response to hypoxia. It is proposed that hypoxia causes reduction in the pH, which in turn increases intracellular iron content by leaching the iron out of iron bound proteins and thus causing induction of ferritins to quench these iron ions [34, 35]. However, we do not know if similar phenomenon occurs in the case of *M*. *tuberculosis* on its exposure to hypoxia.

**Fig 7:**
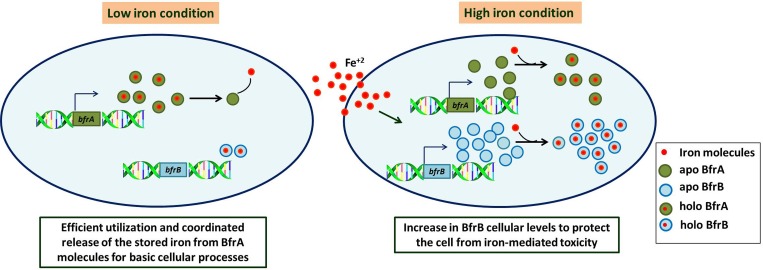
Schematic representation of the proposed functions of BfrA and BfrB of *M*. *tuberculosis*. The figure depicts that under low iron conditions, BfrA is required for efficient utilization of stored iron while BfrB is better equipped to protect the pathogen from oxidative damage in iron rich conditions.

Thus, from our observations it appears that both BfrA and BfrB play an important role in the iron homeostasis of the pathogen, but their roles might not necessarily be completely overlapping. While BfrA seems to be a more preferred protein under low iron conditions, under high iron conditions, BfrB appears to play a more important role. To provide further insight into the properties of these proteins with respect to their commensurate proposed roles, we measured the iron storing and release properties of these proteins and observed that BfrB has relatively much higher capacity for storing iron as compared to BfrA while the latter has a far superior ability to quickly release the stored iron (3 times) (Figs 5A and 6B). These observations suggest that BfrA could be a preferred choice for its role in releasing the iron under low iron conditions in the cell. The quick iron release is indeed facilitated by the presence of heme. Thus, the biochemical properties of these proteins appear to augur well for their proposed functions in the cell (Fig 7).

In conclusion, this study provides another dimension to the existing knowledge in the field of iron storage proteins. Our data provides reasoning for the presence of two ferritins in the same organism and we delineate the functional requirement of these two proteins under varying cellular conditions based on the differences in the iron storage and release properties of BfrA and BfrB. Our findings suggest that bacterioferritins are required for efficient utilization of stored iron under low iron conditions while ferritins are better equipped to prevent the pathogen from oxidative damage in iron rich conditions (Fig 7). This study for the first time also provides insights into the link between ferritins and hypoxic conditions in *M*. *tuberculosis* and suggests the associated mechanism.

## Supporting Information

S1 Fig **Specificity of the anti-BfrA (a) and anti-BfrB (b) polyclonal antiserums.** (a). As shown in the immunoblot, in the lysate derived from *ΔbfrA* mutant (lane 3), anti-BfrA antiserum did not recognize BfrB as there was no band detected near 20 kDa in *ΔbfrA* mutant cell lysate. However, in the lysates derived from *M*. *tuberculosis* H37Rv (lane 1) and *ΔbfrB* mutant (lane 2), BfrA could be recognized. (b). As shown in the immunoblot, in the lysate derived from *ΔbfrB* mutant (lane 2), anti-BfrB antiserum did not recognize BfrA as there was no band detected near 20 kDa in *ΔbfrB* mutant cell lysate. However, in the lysates derived from *M*. *tuberculosis* H37Rv (lane 1) and *ΔbfrA* mutant (lane 3), BfrB could be recognized. M: Protein Marker.
(TIF)

S2 Fig Effect of H_2_O_2_ on the growth of *M*. *tuberculosis*. *M*. *tuberculosis* H37Rv was grown in the minimal media either in absence of H_2_O_2_ (Blue) or in the presence of 0.25 mM H_2_O_2_ (Red).
(TIF)

S3 Fig Purification of Wild type BfrA and its site directed mutant (BfrAM52L). The purified -proteins were analysed for the purity by using electrophoresis on 12.5% SDS-PAG. The figure depicts 20 kDa band of both the proteins.
(TIF)
