# Supplementary figures and images for: Differential Roles of Iron Storage Proteins in Maintaining the Iron Homeostasis in Mycobacterium tuberculosis
Source: PLoS One. 2017 Jan 6;12(1):e0169545. doi: 10.1371/journal.pone.0169545 (PMC5218490; doi:10.1371/journal.pone.0169545)

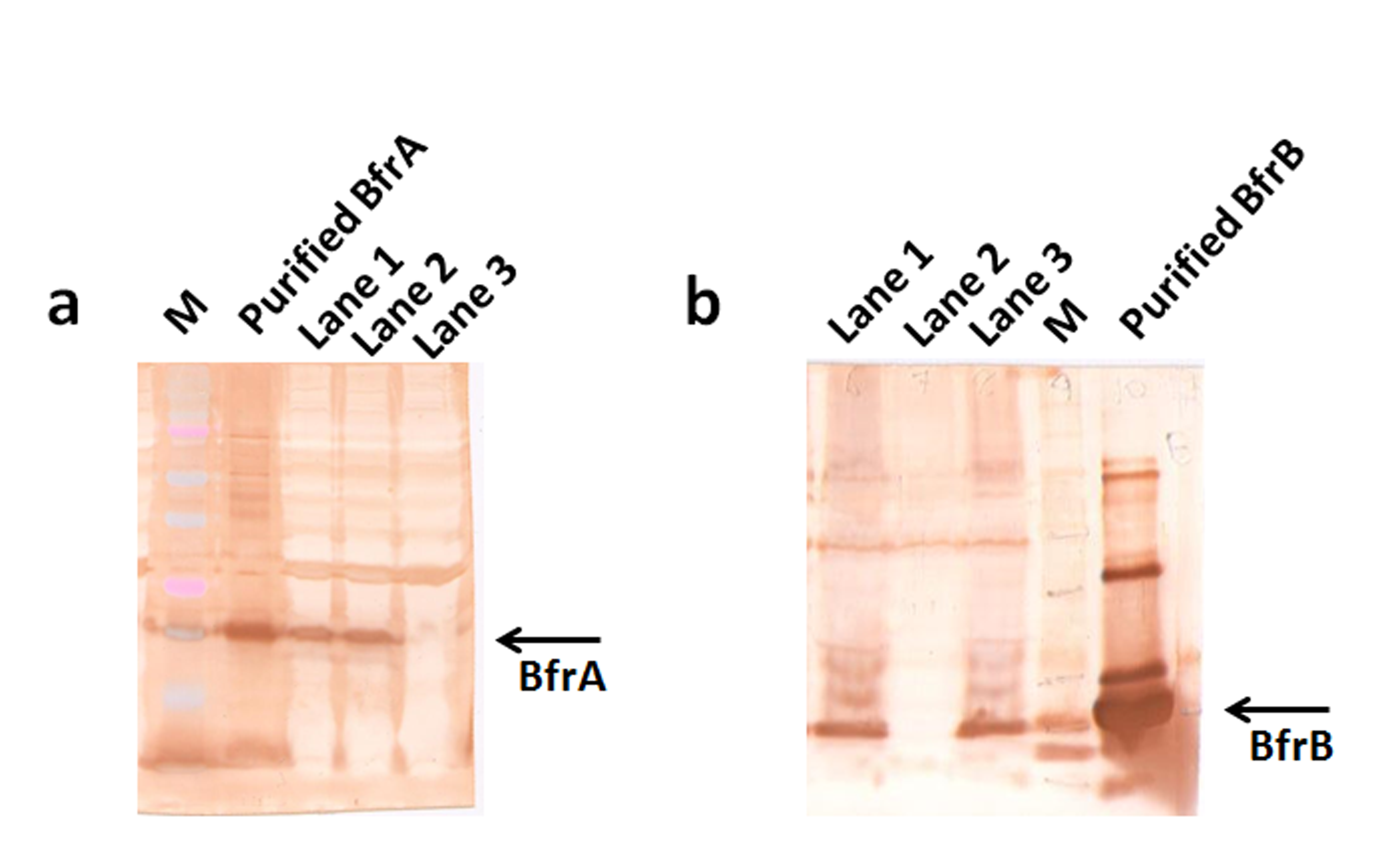

Supplement: S1 Fig — Specificity of the anti-BfrA (a) and anti-BfrB (b) polyclonal antiserums. (a). As shown in the immunoblot, in the lysate derived from ΔbfrA mutant (lane 3), anti-BfrA antiserum did not recognize BfrB as there was no band detected near 20 kDa in ΔbfrA mutant cell lysate. However, in the lysates derived from M. tuberculosis H37Rv (lane 1) and ΔbfrB mutant (lane 2), BfrA could be recognized. (b). As shown in the immunoblot, in the lysate derived from ΔbfrB mutant (lane 2), anti-BfrB antiserum did not recognize BfrA as there was no band detected near 20 kDa in ΔbfrB mutant cell lysate. However, in the lysates derived from M. tuberculosis H37Rv (lane 1) and ΔbfrA mutant (lane 3), BfrB could be recognized. M: Protein Marker. (TIF) [file pone.0169545.s001.tif]

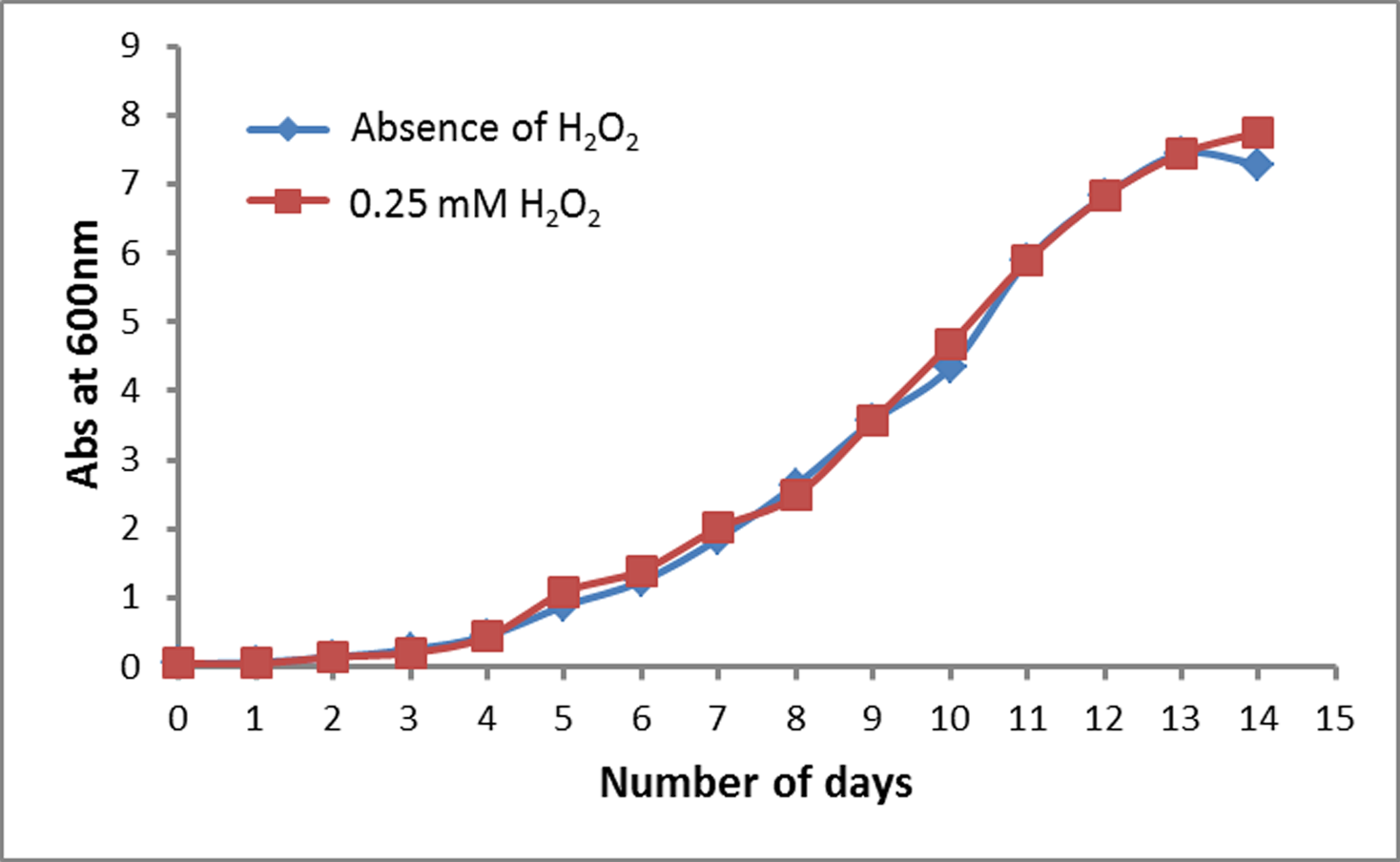

Supplement: S2 Fig — M. tuberculosis H37Rv was grown in the minimal media either in absence of H2O2 (Blue) or in the presence of 0.25 mM H2O2 (Red). (TIF) [file pone.0169545.s002.tif]

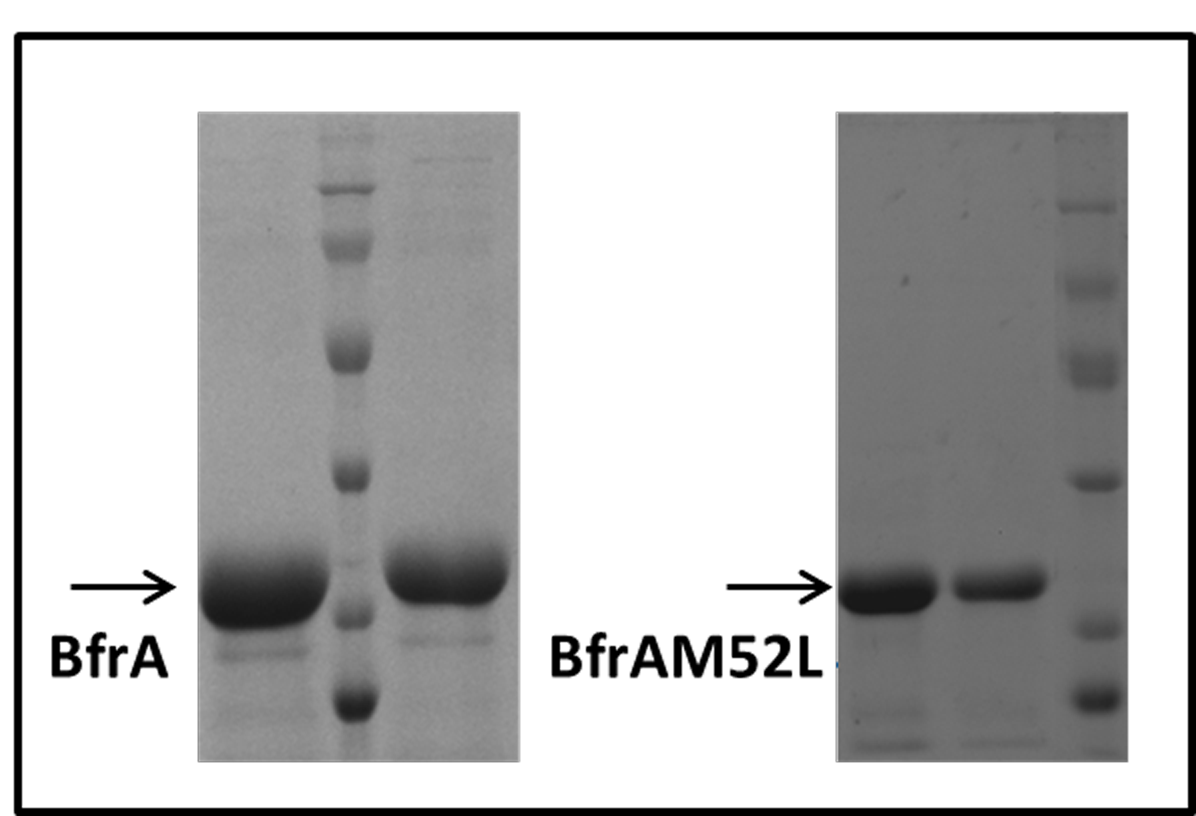

Supplement: S3 Fig — The purified -proteins were analysed for the purity by using electrophoresis on 12.5% SDS-PAG. The figure depicts 20 kDa band of both the proteins. (TIF) [file pone.0169545.s003.tif]
